# Correction: Microbiota-Independent Ameliorative Effects of Antibiotics on Spontaneous Th2-Associated Pathology of the Small Intestine

**DOI:** 10.1371/journal.pone.0133787

**Published:** 2015-07-20

**Authors:** The PLOS ONE Staff

## Notice of Republication

This article was republished on March 20, 2015, to correct an error introduced during the typesetting process. The legend for Figure 4 was misplaced within the Results section of the paper. The publisher apologizes for the errors. Please download this article again to view the correct version. The originally published, uncorrected article and the republished, corrected article are provided here for reference.

## Supporting Information

S1 File Originally published, uncorrected article. (PDF)

S2 File Republished, corrected article. (PDF)
