# Supplementary material for: Correction: Microbiota-Independent Ameliorative Effects of Antibiotics on Spontaneous Th2-Associated Pathology of the Small Intestine
Source: PLoS One. 2015 Jul 20;10(7):e0133787. doi: 10.1371/journal.pone.0133787 (PMC4507877; doi:10.1371/journal.pone.0133787)
Supplement: S2 File — (PDF) [file pone.0133787.s002.pdf]

PLOS | ONE

RESEARCH ARTICLE

Microbiota-Independent Ameliorative Effects of Antibiotics on Spontaneous Th2-Associated Pathology of the Small Intestine

Daehee Han[1,2], Matthew C. Walsh[3], Kwang Soon Kim[1,2], Sung-Wook Hong[1,2], Junyoung Lee[1,2], Jaeu Yi[1,2], Gloriany Rivas[3], Charles D. Surh[1,2,4]*, Yongwon Choi[3]*

1 Academy of Immunology and Microbiology, Institute for Basic Science, Pohang, 790–784, Republic of Korea, **2** Department of Integrative Biosciences and Biotechnology, Pohang University of Science and Technology, Pohang, 790–784, Republic of Korea, **3** Department of Pathology and Laboratory Medicine, University of Pennsylvania Perelman School of Medicine, Philadelphia, Pennsylvania, 19104, United States of America, **4** Division of Developmental Immunology, La Jolla Institute for Allergy and Immunology, La Jolla, California, 92037, United States of America

* ychoi3@mail.med.upenn.edu (YC); csurh@ibs.re.kr (CDS)

Abstract

We have previously generated a mouse model of spontaneous Th2-associated disease of the small intestine called TRAF6ΔDC, in which dendritic cell (DC)-intrinsic expression of the signaling mediator TRAF6 is ablated. Interestingly, broad-spectrum antibiotic treatment ameliorates TRAF6ΔDC disease, implying a role for commensal microbiota in disease development. However, the relationship between the drug effects and commensal microbiota status remains to be formally demonstrated. To directly assess this relationship, we have now generated TRAF6ΔDC bone marrow chimera mice under germ-free (GF) conditions lacking commensal microbiota, and found, unexpectedly, that Th2-associated disease is actually exacerbated in GF TRAF6ΔDC mice compared to specific pathogen-free (SPF) TRAF6ΔDC mice. At the same time, broad-spectrum antibiotic treatment of GF TRAF6ΔDC mice has an ameliorative effect similar to that observed in antibiotics-treated SPF TRAF6ΔDC mice, implying a commensal microbiota-independent effect of broad-spectrum antibiotic treatment. We further found that treatment of GF TRAF6ΔDC mice with broad-spectrum antibiotics increases Foxp3^{+} Treg populations in lymphoid organs and the small intestine, pointing to a possible mechanism by which treatment may directly exert an immunomodulatory effect. To investigate links between the exacerbated phenotype of the small intestines of GF TRAF6ΔDC mice and local microbiota, we performed microbiotic profiling of the luminal contents specifically within the small intestines of diseased TRAF6ΔDC mice, and, when compared to co-housed control mice, found significantly increased total bacterial content characterized by specific increases in *Firmicutes Lactobacillus* species. These data suggest a protective effect of *Firmicutes Lactobacillus* against the spontaneous Th2-related inflammation of the small intestine of the TRAF6ΔDC model, and may represent a potential mechanism for related disease phenotypes.

OPEN ACCESS

Citation: Han D, Walsh MC, Kim KS, Hong S-W, Lee J, Yi J, et al. (2015) Microbiota-Independent Ameliorative Effects of Antibiotics on Spontaneous Th2-Associated Pathology of the Small Intestine. PLoS ONE 10(2): e0118795. doi:10.1371/journal.pone.0118795

Received: November 11, 2014

Accepted: January 15, 2015

Published: February 17, 2015

Copyright: © 2015 Han et al. This is an open access article distributed under the terms of the Creative Commons Attribution License, which permits unrestricted use, distribution, and reproduction in any medium, provided the original author and source are credited.

Data Availability Statement: All relevant data are within the paper.

Funding: This work was supported by the Institute for Basic Science (IBS-R005-D1-2014-a00 to DH, KSK, SH, JL, JY, CDS), the Korea Institute of Oriental Medicine (KIOM), Ministry of Education, Science and Technology (MEST), Korea (K13050 to YC), and the National Institutes of Health (AI064909, AI108627 to YC). The funders had no role in study design, data collection and analysis, decision to publish, or preparation of the manuscript.

Competing Interests: The authors have declared that no competing interests exist.

Introduction

Immune homeostasis along the intestinal tract involves a delicate balance between protective immunity to encountered pathogenic microorganisms and tolerance to commensal gut microbiota and other luminal antigens. Changes in either the microbiotic profile along mucosal borders, or in the manner in which microbiota-associated stimuli are interpreted by host cells can lead to a variety of inflammatory diseases [1]. The intestinal tract is home to a vast array of specialized immune cells as well as absorptive and barrier cells, and the types and functions of these cells change dramatically from the small intestine, where nutrient digestion and absorption occurs in the presence of a relatively low density of commensal microbiota, to the large intestine, where most water re-uptake occurs amongst the highest density and diversity of commensal microbiota found in the body. Because of these anatomic and physiologic differences, inflammatory gut disease phenotypes presently identified are typically categorized according to distinct intestinal regions, even if the specific mechanisms of cellular dysfunction are not currently understood [2]. It is therefore important to develop and characterize animal models that will enable better understanding of both the causes of inflammatory disease in the small versus large intestine, and the role(s) of the interactions between extracellular stimuli, such as commensal microbiota, and intestinal immune cells during the development of disease.

We have recently generated a new mouse model, TRAF6ΔDC [3], in which dendritic cell (DC)-intrinsic expression of the signaling mediator TRAF6 is ablated, resulting in spontaneous development of Th2-associated immune pathology and fibrosis of the small intestine. This pathology is linked to decreased regulatory T cell (Treg) numbers in the small intestines of TRAF6ΔDC mice [3]. The spontaneous nature of TRAF6ΔDC disease development, the Th2-specific phenotype (as opposed to the predominantly Th1 phenotype of Crohn's disease [2]), and its relatively unusual localization to the small rather than large intestine—particularly the proximal small intestine, for which there are no known models of spontaneous inflammation [2]—make TRAF6ΔDC a unique and potentially powerful tool for studying intestinal immune homeostasis. Therefore, more complete characterization of the mechanisms underlying TRAF6ΔDC is required.

Inflammatory phenotypes in the gut are often linked to commensal microbiota [4], and in fact, most conventional models of chronic intestinal inflammation are resolved when mice are maintained under germ-free (GF) conditions, suggesting that stimuli from commensal microbiota are key factors in triggering disease [5,6]. Further, it has also been shown in the SAMP1/YitFc mouse model of Crohn's disease that spontaneous inflammation localized to the small intestine, in which only a minority of the commensal microbiotic population resides, is ameliorated with antibiotics treatment or maintenance under GF conditions [7,8]. Changes in the composition of the commensal microbiota can lead to intestinal inflammation [9]. In our previous efforts, microbiotic profiling of total gut commensals revealed no remarkable disruption of microbiotic homeostasis in TRAF6ΔDC mice; however, broad-spectrum antibiotic treatment of TRAF6ΔDC mice resulted in near complete amelioration of spontaneous immune responses [3]. One possible explanation for this observation is abnormal processing of typical microbiotic stimuli by TRAF6ΔDC intestinal cells, leading to pathogenic immunity. However, it also remains possible that antibiotic treatment has microbiota-independent effects on immune homeostasis that are relevant to the TRAF6ΔDC phenotype, and that this may be an important consideration for studying related disease phenotypes. In fact, other groups have reported that antibiotics may have direct effects on immune homeostasis through modulation of lymphocyte proliferation, cytokine elaboration, antibody production, and/or other ill-defined immunosuppressive mechanisms [10–13]. Also, because most commensal microbiota are found in the large intestine, while TRAF6ΔDC disease localizes to the small intestine, it is

possible that disruption of microbiotic homeostasis may occur regionally in the small intestine, which is a more acidic environment, thereby altering the extracellular signals received by the local immune cells.

To dissect these issues we studied the TRAF6ΔDC model under GF conditions, i.e., in the absence of commensal microbiota. In contrast to our expectation, we found that TRAF6ΔDC gut immune pathology is exacerbated, not ameliorated, under GF conditions. Moreover, this enhanced gut pathology could be ameliorated by broad-spectrum antibiotic treatment, which appears to also enhance gut-associated Tregs in a microbiota-independent manner. Finally, we identified local disruptions of microbiotic homeostasis in the TRAF6ΔDC small intestines, which may suggest protective effects of certain commensal microbiota against spontaneous immune pathology in the small intestine.

Materials and Methods

Animals

TRAF6ΔDC mice were generated by crossing mice carrying floxed alleles of Traf6 with CD11c-Cre transgenic mice [3]. All mice were backcrossed to the C57BL/6 background more than twenty generations. Eight control TRAF6 flx/flx mice (WT, no Cre) and 8 TRAF6ΔDC mice (ΔDC) were used for small intestine bacteria taxonomic analysis and as donors for bone marrow chimeras. Specific pathogen-free (SPF) C57BL/6 mice were purchased from Jackson Laboratory. Mice were maintained under SPF conditions in the animal care facility at POSTECH. A colony of germ-free (GF) C57BL/6 mice was established at POSTECH from breeders obtained from Dr. Andrew Macpherson (Bern Univ., Switzerland). Eighteen SPF C57BL/6 mice and 50 GF C57BL/6 mice were used as recipients for bone marrow chimeras and antibiotic treatment experiments. All mice received care in compliance with the protocols approved by the Institutional Animal Care and Use Committees (IACUC) of the Pohang University of Science and Technology (2013–01–0012). GF mice were maintained in sterile flexible film isolators (Class Biological Clean Ltd., USA) and GF status was monitored monthly by anaerobic and aerobic culture of cecal contents. Normal chow diet (2018S, Harlan Laboratories) and purified water were supplied after autoclaving. All mice were humanely euthanized with CO_2 before analysis or collecting cell populations.

Establishment of Bone Marrow Chimeras

For irradiation of GF mice, the mice were transferred to an autoclaved plastic cylinder sealed with mylar film. The cylinder containing GF recipient mice was irradiated with a lethal dose of 950 cGy using X-RAD 320 (Precision X-ray, USA). After irradiation, the cylinder was connected to the GF isolator and mice were transferred back to the isolator. SPF mice were irradiated in a routine manner with the same dose. Bone marrow stem cells were isolated from tibias and femurs of control TRAF6 flx/flx (WT, no Cre) or TRAF6ΔDC (ΔDC) mice and T cells were removed by employing combined anti-CD4, anti-CD8, and anti-CD90.2 MACS depletion (Miltenyi Biotec). The cells were transferred into autoclaved amber glass vials with silicone caps and imported into GF isolator after sterilizing with 2% peracetic acid solution. 5×10^6 cells were transferred via retro-orbital injection into irradiated GF or SPF C57BL/6 recipient mice. Chimeric mice were used for experiments upon hematopoietic reconstitution 6 to 8 weeks post bone marrow transplantation.

Cell Isolation

Single cells were isolated from spleens and mesenteric lymph nodes by mechanical disruption on 70 μm cell strainers (BD). For the isolation of intestinal lamina propria lymphocytes,

epithelial cells were removed by incubation in stripping buffer containing 3% FBS, 10 mM EDTA, 1 mM sodium pyruvate, and 20 mM HEPES in PBS at 37°C for 15 min in shaker. The remaining pieces were minced and incubated in 3% FBS RPMI-1640 medium containing 1 mg/ml Collagenase D (Roche), 50 μg/ml DNaseI (Sigma), 1 mM sodium pyruvate, 1 mM non-essential amino acid, and 20 mM HEPES at 37°C for 40 min in shaker. Mononuclear cells were collected at the interface between 40% and 75% Percoll (GE Healthcare) after centrifuging at 2,500 rpm at room temperature for 20 min with no brake.

Histological Analysis

Intestinal specimens were cut longitudinally and shaped into inside-out Swiss rolls. Paraffin-embedded sections were performed after fixation in 4% paraformaldehyde solution (Sigma). Routine hematoxylin-eosin staining was performed using hematocylin for nucleus staining, and eosin for cytoplasm and muscle layer.

Quantitative PCR

Tissues were homogenized in TRIzol (Invitrogen) after freezing in liquid nitrogen. cDNAs were established by using QuantiTect Reverse Transcription Kit (QIAGEN). TaqMan gene probes were used with TaqMan Universal PCR Master Mix (Applied Biosystems) and run on ViiATM 7 Real-time PCR System (Applied Biosystems): 2 min at 50°C, 10 min at 95°C, 50 cycles of 15 s 95°C, 1 min at 60°C, and signals were detected during annealing step (60°C). Relative mRNA expression levels of all samples were normalized to 18S mRNA. The TaqMan gene probes (Applied Biosystems) used: Acta2 (Mm01546113_m1), Igf1 (Mm00439560_m1), Il13 (Mm99999190_m1), Il5 (Mm00439646_m1), Il4 (Mm00445259_m1), and 18S ribosomal RNA (Hs99999901_s1).

Buffers and Media

FACS buffer containing 2% FBS and 0.7 mM EDTA was used to prepare, stain, and wash isolated cells. Complete RPMI-1640 medium containing 10% FBS, 10 U/ml Penicillin, 10 mg/ml Streptomycin, 2 mM L-glutamin (Invitrogen), and 50 mM b-mercapotethanol (Sigma) was used for cell culture and stimulation.

Flow Cytometry

For intracellular staining, 10^6 cells were stimulated in round-bottom 96-well plates with complete RPMI1640 medium containing 500 ng/ml ionomycin and 5 ng/ml PMA (Sigma) in the presence of Brefeldin A (BD) for the last 3 hr. After surface staining with fluorochrome-conjugated antibodies including CD4 (RM4–5), CD8 (53–6.7), and CD90.2 (53–2.1), the cells were fixed and permeabilized with fixation/permeabilization reagent (eBioscience) and stained with intracellular protein-specific antibodies specific for IL-13 (eBio13A), IFN-g (XMG1.2), and Foxp3 (FJK-16 s). All stained samples were analyzed on an LSR Fortessa flow cytometer (BD) and the raw data were calculated and visualized with FlowJo software (Tree Star). All of the antibodies were purchased from eBioscience and Tonbo biosciences.

Antibiotic Treatment

Broad-spectrum antibiotic treatment was supplied for 2 weeks in drinking water containing ampicillin (1 g/L, Sigma), vancomycin (0.5 g/L, Calbiochem), neomycin sulfate (1 g/L, Calbiochem), and metronidazole (1 g/L, Sigma). All antibiotic solutions were prepared by sterile by 0.2 μm membrane filtration. The antibiotic solution was refreshed every 4 days.

Small intestinal bacteria taxonomic analysis

Total DNA was isolated from small intestine contents (mostly from ileum) by using the FastDNA SPIN extraction kit (MP Biomedicals, Santa Ana, CA, USA) according to the manufacturer's instructions. The deep sequencing analyses of bacterial composition including DNA amplification, sequencing, and sequence analyses were performed by Chunlab, Inc. (Seoul, Korea) as previously described [14]. Briefly, the target region of the 16S rRNA gene (V1–V3 of variable region) was amplified by using barcoded fusion primers with a C1000 Touch thermal cycler (Bio-Rad, Hercules, CA, USA). The amplified products were purified by the QIAquick PCR purification kit (Qiagen, Valencia, CA, USA) and quantified by the PicoGreen dsDNA Assay kit (Invitrogen, Carlsbad, CA, USA). The equimolar concentration of the amplicon from each sample was pooled and sequencing performed on a Roche/454 GS Junior system according to the manufacturer's instructions.

Quantification of Gut Microbiota

Fecal pellets and small intestinal contents were collected from 8 week-old mice. Some groups were fed by broad-spectrum antibiotic water (Abx) for 2–4 weeks. DNA was isolated from the collected samples by using the PSP Spin Stool DNA Kit (Stratec molecular). Q-PCR was performed with 16S rDNA probe. Copy numbers of bacteria were determined according to standard curves calculated from serially diluted *E. coli*. plasmid templates.

Statistics

Data were analyzed by using Prism software (GraphPad) with an unpaired or paired Student's *t* test. *P* values less than 0.05 were considered significant.

Ethics Statement

This research was approved by the Institutional Animal Care and Use Committees (IACUC) of the Pohang University of Science and Technology (2013–01–0012). Mouse care and experimental procedures were performed in accordance with all institutional guidelines for the ethical use of non-human animals in research and protocols from IACUC of the Pohang University of Science and Technology.

Results

Germ-free conditions exacerbate Th2-associated immune pathology in TRAF6ΔDC small intestine

We have previously shown that broad-spectrum antibiotic treatment ameliorates spontaneous Th2-associated disease in TRAF6ΔDC mice. However, the relationship between the drug effects and commensal microbiota status remains unclear. Therefore, we have established TRAF6ΔDC bone marrow chimera mice under GF conditions. We anticipated that GF TRAF6ΔDC mice would recapitulate the effects of antibiotic treatment since antibiotics are widely used to mimic GF conditions by broadly ablating gut commensal bacteria. Contrary to our expectation, GF TRAF6ΔDC bone marrow chimeras did not phenocopy antibiotic-treated TRAF6ΔDC specific pathogen-free (SPF) mice, but instead exhibited exacerbated gut phenotypes including gross appearance (Fig. 1A) and histology (Fig. 1B), as well as increased Th2 cytokine-producing cells (Fig. 2A and B). Elevated serum IgE levels (Fig. 2C) in GF TRAF6ΔDC bone marrow chimeras were consistent with observations of exacerbated gut inflammation. Furthermore, small intestine tissue gene expression levels of pro-fibrotic markers (Acta2 and Igf-1) and Th2 cytokines

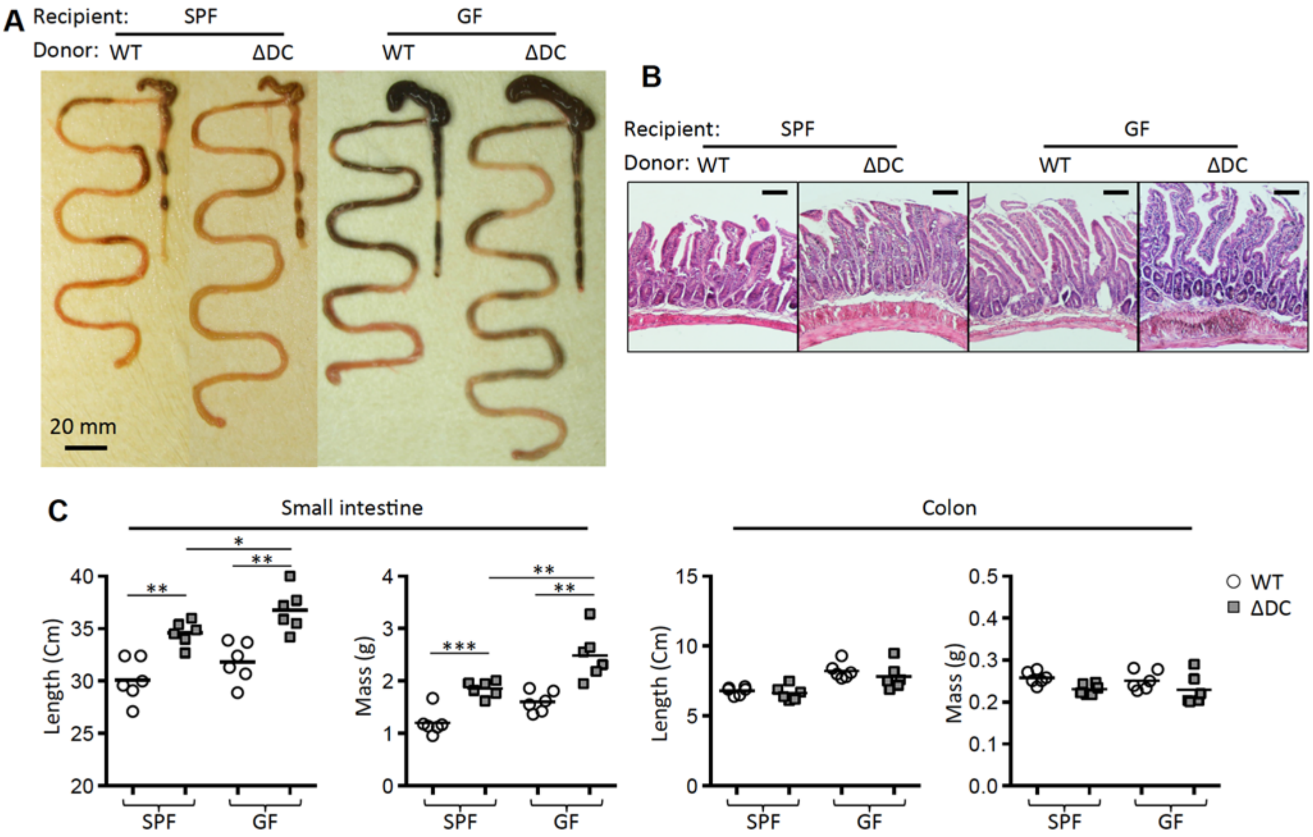

Fig 1. Germ-free conditions exacerbate inflammation of TRAF6ΔDC small intestine. (A) Representative gut images from control (WT) and TRAF6ΔDC (ΔDC) bone marrow chimera established in specific pathogen free (SPF) or germ-free (GF) condition at 8 weeks post-reconstitution. (B) Histological analyses were performed by H&E staining of duodenum from each mice. Scale bars represent 100 μm. (C) Lengths and masses of small intestines and colons were measured in control (WT) and TRAF6ΔDC (ΔDC) bone marrow chimera (n = 6). SPF, specific pathogen free; GF, germ-free. *p < 0.05; **p < 0.01; ***p < 0.001; ****p < 0.0001.

doi:10.1371/journal.pone.0118795.g001

(IL-13, IL-5, and IL-4) were increased in GF TRAF6ΔDC bone marrow chimeras compared to SPF TRAF6ΔDC mice (Fig. 2D). Together, these findings imply that microbiota may actually play some positive role in maintain gut immune homeostasis in TRAF6ΔDC mice.

Broad-spectrum antibiotics ameliorate TRAF6ΔDC immune pathology exacerbated by GF conditions

The exacerbated disease phenotype observed in GF TRAF6ΔDC bone marrow chimeras suggested that antibiotics-mediated disease amelioration might not be microbiota-dependent. To determine if antibiotics function independently of gut microbiota in the context of immune pathology in the TRAF6ΔDC model, broad-spectrum antibiotics were administered orally to GF TRAF6ΔDC bone marrow chimeras at 6 weeks post-reconstitution for two weeks prior to phenotyping. Interestingly, Th2 cytokine-producing cells were reduced in GF TRAF6ΔDC bone marrow chimeras when treated with antibiotics (Fig. 3A and B). Length and mass of small intestines were reduced in antibiotics-treated GF TRAF6ΔDC mice (Fig. 3C), as were small intestine gene expression levels of both fibrotic markers and Th2 cytokines (Fig. 3D). These data suggest that broad-spectrum antibiotics may directly suppress TRAF6ΔDC Th2-associated gut inflammation in a manner independent of gut microbiota.

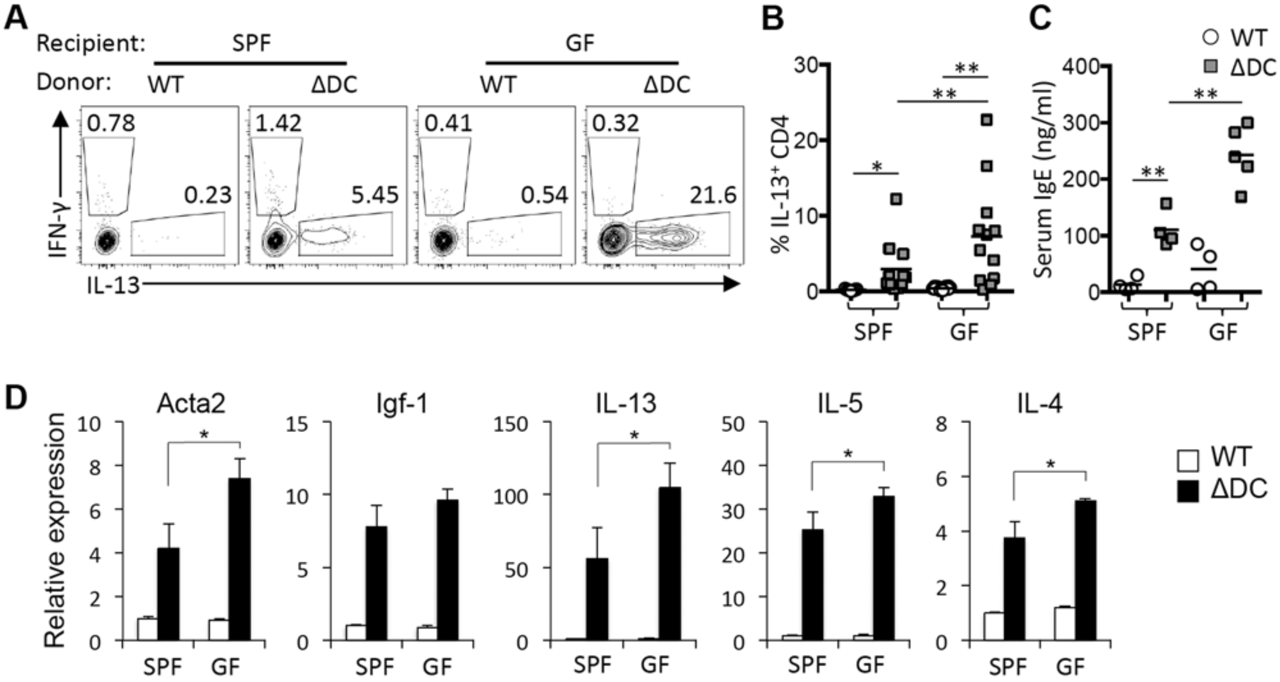

Fig 2. Germ-free conditions exacerbate Th2-associated immune pathology in TRAF6ΔDC small intestine. (A and B) Increases of IL-13+ cells in mesenteric lymph nodes of TRAF6ΔDC (ΔDC) bone marrow chimera in germ-free (GF) condition compared to specific pathogen free (SPF) condition. The representative FACS plots gated on CD4 T cells of mesenteric lymph nodes show intracellular staining for interferon-γ (IFN-γ) and IL-13. **(C)** Serum immunoglobulin levels in the chimera were measured by ELISA. **(D)** Fibrosis markers (Acta2 and Igf-1) and Th2 cell cytokines (IL-13, IL-5, and IL-4) mRNA expression levels in the ileum region of small intestines from SPF or GF control and TRAF6Δ bone marrow chimera. Histograms (mean ± SD) are representative of three independent experiments. $^*p < 0.05$; $^{**}p < 0.01$.

doi:10.1371/journal.pone.0118795.g002

Increased Tregs in GF TRAF6ΔDC lymphoid organs following antibiotic treatment

We have previously reported rescue of the small intestine Treg population in TRAF6ΔDC mice treated with broad-spectrum antibiotics [3]. Therefore, we hypothesized that antibiotic treatment of GF TRAF6ΔDC mice would similarly enhance the Treg population. Since we cannot isolate cells from TRAF6ΔDC small intestines once severe fibrosis sets in, spleens and mesenteric lymph nodes were analyzed instead (Fig. 4A and B). Increased Treg cells were observed in GF TRAF6ΔDC lymphoid organs at 8 weeks post-reconstitution after antibiotic water treatment for last two weeks (Fig. 4A and B). Interestingly, we also observed increased Treg numbers in control GF mice receiving similar antibiotics treatment (Fig. 4C). These findings suggest a general direct positive microbiota-independent effect of broad-spectrum antibiotic treatment on Treg populations, either by recruitment and/or proliferation, in the lymphoid organs and peripheral tissues. Further, this effect may be relevant to maintaining immune homeostasis of the TRAF6ΔDC small intestine.

TRAF6ΔDC mice exhibit disruption of microbiotic homeostasis localized to the small intestine

We previously reported that there are no apparent differences in the microbiotic profiles of fecal material collected from TRAF6ΔDC mice and co-housed control mice. To better understand why GF conditions exacerbated the pathologic phenotype of TRAF6ΔDC mice, we performed microbiotic profiling of the luminal contents specifically within the small intestines of diseased

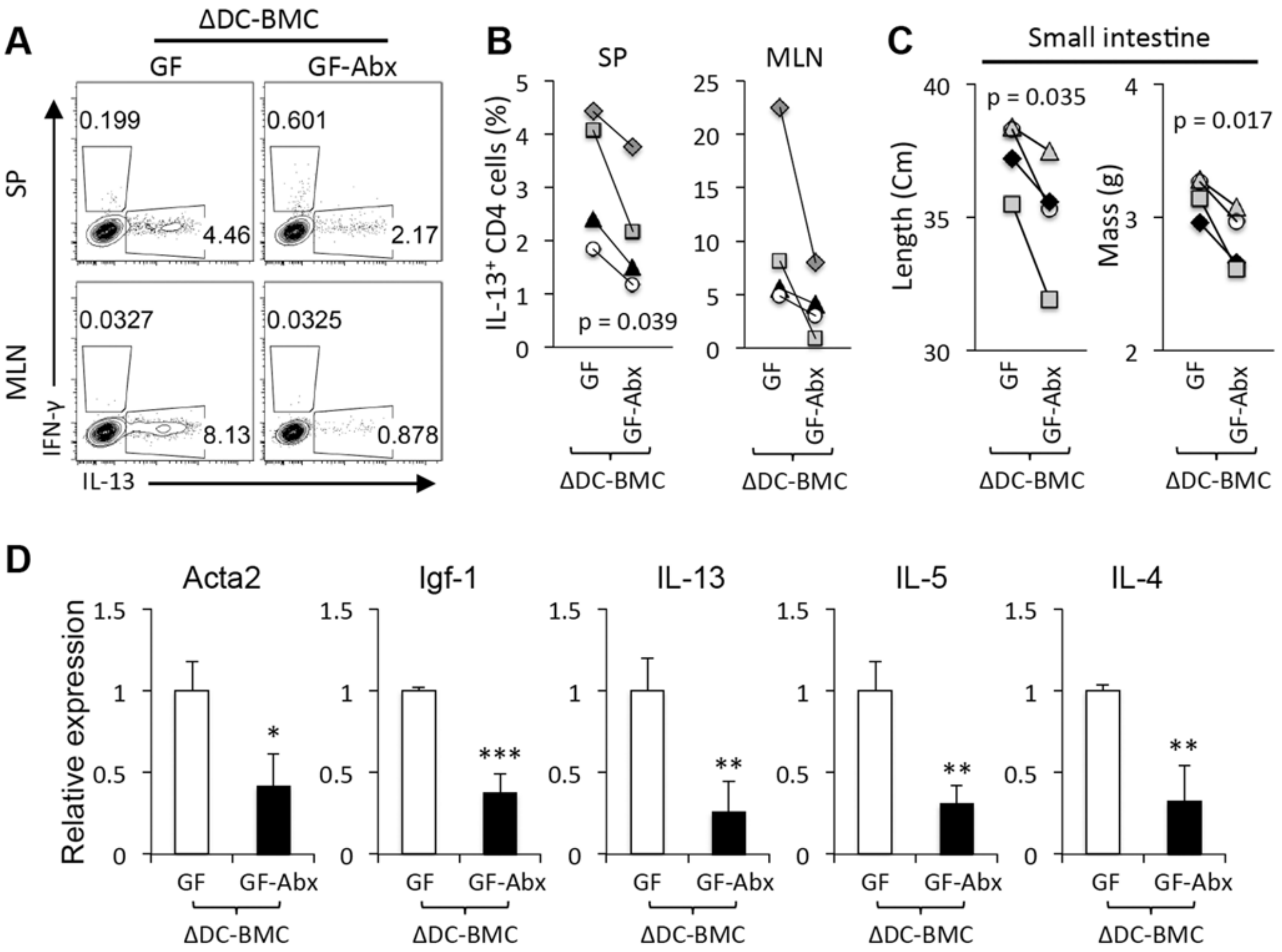

Fig 3. Broad-spectrum antibiotics ameliorate TRAF6ΔDC immune pathology exacerbated by GF conditions. (A) FACS plots gated on $CD4^+$ T cells show intracellular staining for interferon-γ (IFN-γ) and IL-13 from each indicated organ of TRAF6ΔDC bone marrow chimera (ΔDC-BMC) in germ-free (GF) condition at 8 weeks post-reconstitution. Some of TRAF6ΔDC bone marrow chimeras (ΔDC) were provided with broad-spectrum antibiotic water (Abx). The antibiotic water, containing 1 g/L Ampicillin, 1 g/L Neomycin, 0.5 g/L Vancomycin, and 1 g/L Metronidazole, was provided ad libitum in water to TRAF6ΔDC bone marrow chimera (ΔDC) for last 2 weeks. **(B)** Percentage of IL-13^+ CD4 T cells were shown in each organs of TRAF6ΔDC bone marrow chimera (ΔDC-BMC) in germ-free (GF) or germ-free under antibiotics (GF-Abx). **(C)** Lengths and masses of small intestines were measured in GF and GF-Abx TRAF6ΔDC bone marrow chimera (ΔDC-BMC). **(D)** Fibrosis markers (Acta2 and Igf-1) and Th2 cell cytokines (IL-13, IL-5, and IL-4) mRNA expression levels in the ileum region of small intestines from GF and GF-Abx TRAF6ΔDC bone marrow chimera (ΔDC-BMC). Histograms (mean ± SD) are representative of four independent experiments. SP, spleen; MLN, mesenteric lymph node. *$p < 0.05$; **$p < 0.01$; ***$p < 0.001$.

doi:10.1371/journal.pone.0118795.g003

TRAF6ΔDC mice or co-housed control mice. Total genomic DNA was isolated from small intestine luminal contents in ileal sections of control and TRAF6ΔDC mice, and metagenomic analyses were performed to determine any differences in bacterial communities. Interestingly, the bacterial composition of the TRAF6ΔDC small intestine differs from control, specifically with respect to the phylum Firmicutes, which was dramatically increased, while Bacteroidetes was decreased (Fig. 5A). Heatmap analyses further details these differences (Fig. 5B), showing strongly increased *Lactobacillus* in the TRAF6ΔDC small intestine compared to other bacteria. To clarify the factors leading to altered bacterial composition, total bacterial copy number was quantified using PCR for bacterial 16S rRNA (Fig. 5C). Even though relative copy number of bacterial 16S rRNA in the small intestine is much lower than for colon contents or feces, significant increases in copy number in TRAF6ΔDC small intestines were observed. Taken together, dramatically

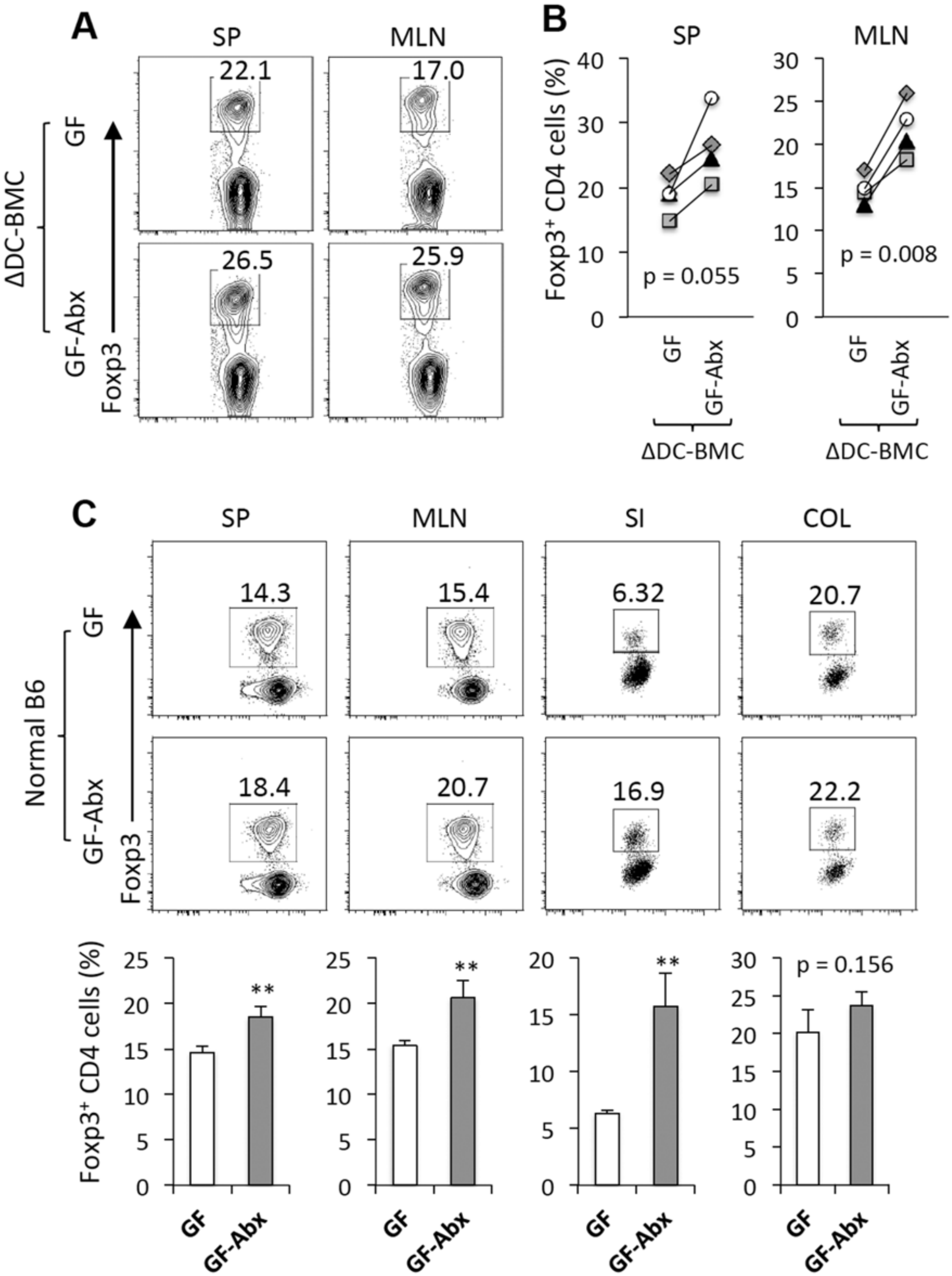

Fig 4. Increased Tregs in GF TRAF6ΔDC lymphoid organs following antibiotic treatment. (A) FACS plots gated on $CD4^+$ T cells show intracellular staining Foxp3 from each indicated organ of germ-free (GF) TRAF6ΔDC bone marrow chimera (ΔDC-BMC) at 8 weeks post-reconstitution. Broad-spectrum antibiotic water was provided to a group of TRAF6ΔDC bone marrow chimeras (ΔDC) for the last 2 weeks. **(B)** Percentage of $Foxp3^+$ CD4 T cells were determined from the indicated organs of TRAF6ΔDC bone marrow chimera (ΔDC-BMC) in germ-free (GF) or germ-free under antibiotics (GF-Abx). **(C)** Representative FACS plots show $Foxp3^+$ CD4 T cells from each indicated organ of germ-free (GF) B6 mice. Some of the mice were provided with broad-spectrum antibiotic water (GF-Abx) for last 2 weeks. Percentage of $Foxp3^+$ CD4 T cells were shown in each organs of germ-free B6 mice with (GF-Abx) or without antibiotics (GF). SP, spleen; MLN, mesenteric lymph node; SI, small intestine; COL, colon. $^{**}p < 0.01$.

doi:10.1371/journal.pone.0118795.g004

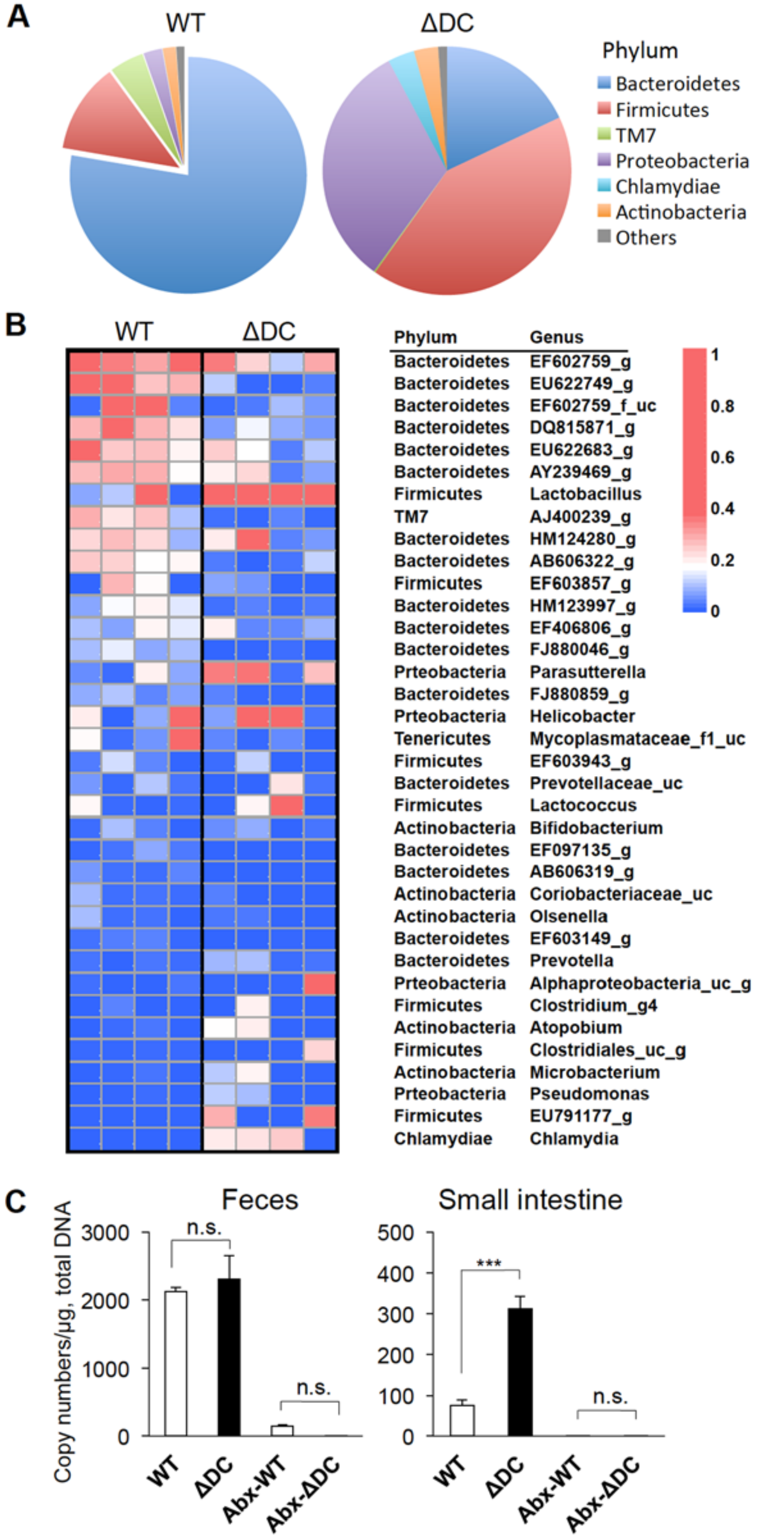

Fig 5. TRAF6ΔDC mice exhibit disruption of microbiotic homeostasis localized to the small intestine. **(A)** Pie charts represent bacterial composition categorized by phylum in the small bowl of control (WT) or TRAF6ΔDC (ΔDC) mice. **(B)** Heatmap analysis shows relative abundance of taxa as a percentage of total 16S rRNA, organized according to genus or most specific assigned taxon. Color scales reflect proportion contributed by each taxon. Total bacterial genome was isolated from small intestinal contents of control (WT)

or TRAF6ΔDC (ΔDC) mice (n = 4, 20 week old littermate and co-housed group). **(C)** The copy number of total bacterial 16S rRNA bacteria from fecal or small intestine contents of control (WT) and TRAF6ΔDC (ΔDC) was measured by comparing to reference E. coli 16S rRNA plasmids. Some groups were fed by broad-spectrum antibiotic water (Abx) for last 2 weeks before collecting samples. **$p < 0.001$; n.s., not significant. Data were analyzed by Anova on Prism software (n = 3).

doi:10.1371/journal.pone.0118795.g005

increased bacterial quantity in TRAF6ΔDC small intestines is explained by elevation of the phylum Firmicutes, especially the genus *Lactobacillus*, which may imply a protective effect of *Firmicutes Lactobacillus* in the context of controlling TRAF6ΔDC Th2-associated disease.

Discussion

Inflammatory autoimmune and allergic diseases of the intestine are of great interest both because of their clinical prevalence and because they result from manifold triggers and underlying causes, most of which are not fully understood. There is also great variability in disease manifestation depending on specific anatomic location, types and activation status of immune cells involved, and lastly, but importantly, how host intestinal cells interact with commensal microbiota of the gut. Because of these many variables, it has been important to develop and characterize robust animal models that accurately represent specific human diseases. In this study we have substantially clarified the host-microbiota relationship and causes of spontaneous small intestine immune pathology in the TRAF6ΔDC mouse model. Specifically, and most importantly, we have shown that broad-spectrum antibiotic treatment of TRAF6ΔDC mice does not primarily exert its disease ameliorating effects by ablating gut microbiota. We have further shown that antibiotic treatment may, under certain circumstances, increase Treg numbers and/or recruitment, which differs from other studies of the effects of antibiotics that focus on colonic Treg cells [15]. Because TRAF6ΔDC is a model based on altering DC function, it may be useful in the future to investigate the direct role of antibiotics treatment on DC function in order to understand how the TRAF6ΔDC phenotype manifests and to better recognize what effects antibiotics may have on DC function in a clinical setting. Additionally, given our findings regarding modulation of Treg status by antibiotics in the absence of microbiota, we must determine whether ablation of the Treg population by antibody or genetic approaches causes re-establishment of any or all components of disease in antibiotics-treated SPF and/or GF TRAF6ΔDC mice. This would further confirm how broad-spectrum antibiotics regulate immune function independently of microbiota in the small intestine.

We determined that GF conditions exacerbated TRAF6ΔDC disease, and by further finding increased *Lactobacillus* localized to the small intestines of TRAF6ΔDC mice, we speculate that these strains provide a protective effect against TRAF6ΔDC inflammatory disease since their removal under GF conditions actually exacerbates disease. In order to confirm this protective effect, it will be important to generate gnotobiotic TRAF6ΔDC mice that specifically contain *Firmicutes Lactobacillus* strains to interrogate whether the GF-enhanced disease phenotype in TRAF6ΔDC mice is ameliorated. *Lactobacillus* species have been well known to possess anti-inflammatory properties in the context of commensal microbiota homeostasis of the large intestine [16], and have been employed in the prevention of inflammatory bowel disease [17,18]. While the levels of commensal bacteria are at least 1000 fold higher in the large intestine than the small intestine, where TRAF6ΔDC disease occurs, significant levels of colonization are found in the ileum, where counts can be as high as 10^8 CFU/mL [1,19]. It is also notable that under inflammatory conditions, concentrations of reactive oxygen species in the gut are often increased [20], creating a more aerobic environment, which may affect the composition of the gut microbiota. *Lactobacillus* species, though anaerobic, can survive well under aerobic

conditions and have a relatively high tolerance for reactive oxygen species, such as hydrogen peroxide (H_2O_2) [21]. The increased levels of *Lactobacillus* detected in the TRAF6ΔDC small intestine raise questions as to whether changes in TRAF6ΔDC microbiotic composition are a reaction to incipient inflammation, or rather occur in parallel due to altered biology of TRAF6-deficient DCs in the gut. For instance, there is reported evidence that TLR signaling in macrophages regulates reactive oxygen species production in a TRAF6-dependent manner [22]. It may be useful to determine whether TRAF6ΔDC small intestinal DCs have a higher propensity for producing reactive oxygen species that may alter gut microbiota composition, and further, whether directly manipulating oxygenation of the TRAF6ΔDC gut affects microbiotic composition and/or inflammatory disease of the small intestine.

The fact that we observed exacerbated inflammatory disease in GF versus SPF TRAF6ΔDC mice suggests that regardless of the specific direct immunomodulatory effects of broad-spectrum antibiotics treatment, or the potential immunosuppressive role of *Lactobacillus*, the trigger and/or antigenic driver of TRAF6ΔDC inflammatory disease is microbiota-independent. While this finding was not predicted given previous results using only broad-spectrum antibiotics under SPF conditions, it may not be surprising given the relatively low bacterial load in the small intestine (compared to the large intestine) where disease is localized. Therefore, in order to fully characterize the nature of inflammatory disease in the TRAF6ΔDC model, we must consider alternative triggers of spontaneous immune pathology in TRAF6ΔDC mice. Because of the key role of the small intestine in digesting and absorbing dietary material, it is possible that disease is triggered by food-related antigen. Moreover, the current work also suggests that microbiota interactions with foodstuff are not required for disease induction. In either case, these possibilities must be further investigated, for instance by modulating dietary intake of TRAF6ΔDC mice under GF and SPF conditions, because a positive finding may identify TRAF6ΔDC as an extremely valuable genetic model for food antigen-related immune pathology and/or allergy.

Acknowledgments

We thank T. Kim and J. Seo for maintenance of the germ-free mouse facility, and H. Jung for assistance with FACS analysis.

Author Contributions

Conceived and designed the experiments: DH MCW CDS YC. Performed the experiments: DH KSK SH JL JY GR. Analyzed the data: DH MCW CDS YC. Wrote the paper: DH MCW CDS YC.

References

1. Koboziev I, Reinoso Webb C, Furr KL, Grisham MB. Role of the enteric microbiota in intestinal homeostasis and inflammation. Free Radic Biol Med. 2014; 68: 122–133. doi: 10.1016/j.freeradbiomed.2013.11.008 PMID: 24275541
2. Mowat C, Cole A, Cole A, Windsor A, Ahmad T, Arnott I, Driscoll R, et al. Guidelines for the management of inflammatory bowel disease in adults. Gut. 2011; 60: 571–607. doi: 10.1136/gut.2010.224154 PMID: 21464096
3. Han D, Walsh MC, Cejas PJ, Dang NN, Kim YF, Kim J, et al. Dendritic cell expression of the signaling molecule TRAF6 is critical for gut microbiota-dependent immune tolerance. Immunity. 2013; 38: 1211–1222. doi: 10.1016/j.immuni.2013.05.012 PMID: 23791643
4. Maloy KJ, Powrie F. Intestinal homeostasis and its breakdown in inflammatory bowel disease. Nature. 2011; 474: 298–306. doi: 10.1038/nature10208 PMID: 21677746
5. Morrissey PJ, Charrier K. Induction of wasting disease in SCID mice by the transfer of normal CD4+/CD45RBhi T cells and the regulation of this autoreactivity by CD4+/CD45RBlo T cells. Res Immunol. 1994; 145: 357–362. PMID: 7701115

6. Strober W 1, Fuss IJ, Blumberg RS. The immunology of mucosal models of inflammation. Annu Rev Immunol. 2002; 20: 495–549. PMID: 11861611
7. Bamias G 1, Marini M, Marini M, Moskaluk CA, Odashima M, Ross WG, Rivera-Nieves J, et al. Down-regulation of intestinal lymphocyte activation and Th1 cytokine production by antibiotic therapy in a murine model of Crohn's disease. J Immunol. 2002; 169: 5308–5314. PMID: 12391251
8. Matsumoto S, Okabe Y, Setoyama H, Takayama K, Ohtsuka J, Funahashi H, et al. Inflammatory bowel disease-like enteritis and caecitis in a senescence accelerated mouse P1/Yit strain. Gut. 1998; 43: 71–78. PMID: 9771408
9. Reading NC, Kasper DL. The starting lineup: key microbial players in intestinal immunity and homeostasis. Front Microbiol. 2011; 2: 148. doi: 10.3389/fmicb.2011.00148 PMID: 21779278
10. Hauser WE Jr., Remington JS. Effect of Antibiotics on the Immune Response. Am J Med. 1982; 72: 711–716. PMID: 7044118
11. Van Vlem B 1, Vanholder R, De Paepe P, Vogelaers D, Ringoir S. Immunomodulating effects of antibiotics: literature review. Infection. 1996; 24: 275–291. PMID: 8875279
12. Morikawa K, Oseko F, Morikawa S, Iwamoto K. Immunomodulatory effects of three macrolides, midecamycin acetate, josamycin, and clarithromycin, on human T-lymphocyte function in vitro. Antimicrob Agents Chemother. 1994; 38: 2643–2647. PMID: 7532933
13. Roszkowski W, Ko HL, Roszkowski K, Jeljaszewicz J, Pulverer G. Antibiotics and immunomodulation: Effects of cefotaxime, amikacin, mezlocillin, piperacillin and clindamycin. Med Microbiol Immunol. 1985; 173: 279–289. PMID: 3969058
14. Kim BS, Song MY, Kim H. The anti-obesity effect of Ephedra sinica through modulation of gut microbiota in obese Korean women. J Ethnopharmacol. 2014; 152: 532–539. doi: 10.1016/j.jep.2014.01.038 PMID: 24556223
15. Atarashi K, Tanoue T, Shima T, Imaoka A, Kuwahara T. Momose Y, et al. Induction of colonic regulatory T cells by indigenous Clostridium species. Science. 2011; 331: 337–341. doi: 10.1126/science.1198469 PMID: 21205640
16. Bindels LB, Beck R, Schakman O, Martin JC, De Backer F, Sohet FM, et al. Restoring specific lactobacilli levels decreases inflammation and muscle atrophy markers in an acute leukemia mouse model. PLoS One. 2012; 7: e37971. doi: 10.1371/journal.pone.0037971 PMID: 22761662
17. Yan F, Cao H, Cover TL, Washington MK, Shi Y, Liu L, et al. Colon-specific delivery of a probiotic-derived soluble protein ameliorates intestinal inflammation in mice through an EGFR-dependent mechanism. J Clin Invest. 2011; 121: 2242–2253. doi: 10.1172/JCI44031 PMID: 21606592
18. Ouwehand AC, Salminen S, Isolauri E. Probiotics: An overview of beneficial effects. Antonie van Leeuwenhoek. 2002; 82: 279–289. PMID: 12369194
19. Quigley EMM, Quera R. Small Intestinal Bacterial Overgrowth: Roles of Antibiotics, Prebiotics, and Probiotics. Gastroenterology. 2006; 130: S78–S90. PMID: 16473077
20. Rezaie A, Parker RD, Abdollahi M. Oxidative stress and pathogenesis of inflammatory bowel dieases: and epiphenomenon or the cause? Dig Dis Sci. 2007; 52: 2015–2021. PMID: 17404859
21. Lin PW, Myers LE, Myers LE, Ray L, Song SC, Nasr TR, Berardinelli AJ, et al. *Lactobacillus rhamnosus* blocks inflammatory signaling in vivo via reactive oxygen species generation. Free Radic Biol Med. 2009; 47: 1205–1211. doi: 10.1016/j.freeradbiomed.2009.07.033 PMID: 19660542
22. West AP, Brodsky IE, Rahner C, Woo DK, Erdjument-Bromage H, Tempst P, et al. TLR signalling augments macrophage bactericidal activity through mitochondrial ROS. Nature. 2011; 472: 476–480. doi: 10.1038/nature09973 PMID: 21525932
